# Supplementary material for: Perceptions of pandemic resume gaps: Survey experimental evidence from the United States
Source: PLoS One. 2023 Mar 16;18(3):e0281449. doi: 10.1371/journal.pone.0281449 (PMC10019729; doi:10.1371/journal.pone.0281449)
Supplement: S1 File — (DOCX) [file pone.0281449.s001.docx]

**Supporting Information**

"Perceptions of Pandemic Resume Gaps: Survey Experimental Evidence from the United States"

Regina Bateson

Graduate School of Public and International Affairs, University of Ottawa

Jan. 27, 2023

Prepared for publication in *PLOS One*

SI Table 1. Hiring scenario vignettes

SI Table 2. Profiles of fictional job applicants for scenario 1

SI Table 3. Profiles of fictional job applicants for scenario 2

SI Table 4. Distribution of randomly assigned employment histories

SI Table 5. Distribution of randomly assigned gender identities

SI Table 6. Distribution of randomly assigned racial identities

SI Table 7. Adjectives provided to survey respondents

SI Table 8. Fig 1 results in table form, with robustness checks

SI Table 9. Fig 2 results in table form, attributes 1-6

SI Table 10. Fig 2 results in table form, attributes 7-11

SI Table 11. Fig 3 results in table form

SI Table 12. Fig 4 results in table form, attributes 1-6

SI Table 13. Fig 4 results in table form, attributes 7-11

SI Table 14. Fig 5 results in table form

SI Table 15. Fig 6 results in table form

SI Table 16. Fig 7 results in table form

SI Table 17. Fig 8 results in table form

**SI Table 1.** Hiring scenario vignettes

| Hiring scenario 1 | With more people vaccinated, many restaurants are re-opening for in-person dining.  Imagine that you are hiring a server for a restaurant in your town or city.  [View profiles of Applicants A, B, and C]  Who would you hire as your restaurant's new server? |
| --- | --- |
| Hiring scenario 2 | As the COVID-19 pandemic wanes, many hotels are resuming normal operations and hiring staff.  Imagine that you are hiring a front desk clerk for a hotel in your town or city.  [View profiles of Applicants D, E, and F]  Who would you hire as your hotel's new front desk clerk? |

**SI Table 2.** Profiles of fictional job applicants for scenario 1, with randomized text in brackets and italics

| **Applicant A** is a *[Black/white] [man/woman]* who is 33 years old. *[He/she]* has 3 children. *[Most recently / until last week]*, *[he/she]* worked as a restaurant server. *[He/she]* has 1 year of college education and 12 years of relevant work experience. Before tips, *[his/her]* most recent wage was $9/hour.  *[ [He/she] was laid off in April 2020, and [he/she] has not worked since. / [He/she] was laid off in April 2020, rehired in June 2020, and laid off again in October 2020. [He/she] has not worked since. / [He/she] was employed continuously throughout the pandemic, until the restaurant where [he/she] worked went out of business a few days ago. / [He/she] stopped working in April 2020 to supervise online school for [his/her] children, and [he/she] has not worked since. ]* | **Applicant B** is a *[Black/white] [man/woman]* who is 28 years old. *[He/she]* has 2 children. *[Most recently / until last week]*, *[he/she]* worked as a bartender. *[He/she]* has a high school diploma and 8 years of relevant experience. Before tips, *[his/her]* most recent wage was $11/hour.  [ *[He/she] was laid off in April 2020, and [he/she] has not worked since.* ***/*** *[He/she] was laid off in April 2020, rehired in June 2020, and laid off again in October 2020.* *[He/she] has not worked since.* ***/*** *[He/she] was employed continuously throughout the pandemic, until the restaurant where [he/she] worked went out of business a few days ago.* ***/*** *[He/she] stopped working in April 2020 to supervise online school for [his/her] children, and [he/she] has not worked since. ]* | **Applicant C** is a *[Black/white] [man/woman]* who is 26 years old. *[He/she]* has 2 children. *[ Most recently / until last week]*, *[he/she]* worked as a restaurant server. *[He/she]* has 2 years of college education and 4 years of relevant work experience. Before tips, *[his/her]* most recent wage was $10/hour.  *[ [He/she] was laid off in April 2020, and [he/she] has not worked since. / [He/she] was laid off in April 2020, rehired in June 2020, and laid off again in October 2020. [He/she] has not worked since. / [He/she] was employed continuously throughout the pandemic, until the restaurant where [he/she] worked went out of business a few days ago. / [He/she] stopped working in April 2020 to supervise online school for [his/her] children, and [he/she] has not worked since. ]* |
| --- | --- | --- |

**SI Table 3.** Profiles of fictional job applicants for scenario 2, with randomized text in brackets and italics

| **Applicant D** is a *[Black/white]* *[man/woman]* who is 32 years old. *[He/she]* has 2 children. *[Most recently / Until last week]*, *[he/she]* worked as a hotel concierge. *[He/she]* has a high school diploma and 8 years of relevant work experience. *[His/her]* most recent wage was $17/hour. *[ [He/she] was laid off in April 2020, and [he/she] has not worked since.* ***/*** *[He/she] was laid off in April 2020, rehired in July 2020, and laid off again in November 2020. [He/she] has not worked since.* ***/*** *[He/she] was employed continuously throughout the pandemic, until the hotel where [he/she] worked went out of business a few days ago.* ***/*** *[He/she] stopped working in April 2020 to supervise online school for [his/her] children, and [he/she] has not worked since. ]* | **Applicant E** is a *[Black/white]* *[man/woman]* who is 35 years old. *[He/she]* has 3 children. *[Most recently / Until last week]*, *[he/she]* worked as a hotel office assistant. *[He/she]* has 1 year of college education and 11 years of relevant work experience. *[His/her]* most recent wage was $22/hour. *[ [He/she] was laid off in April 2020, and [he/she] has not worked since.* ***/*** *[He/she] was laid off in April 2020, rehired in July 2020, and laid off again in November 2020. [He/she] has not worked since.* ***/*** *[He/she] was employed continuously throughout the pandemic, until the hotel where [he/she] worked went out of business a few days ago.* ***/*** *[He/she] stopped working in April 2020 to supervise online school for [his/her] children, and [he/she] has not worked since. ]* | **Applicant F** is a *[Black/white]* *[man/woman]* who is 29 years old. *[He/she]* has 2 children. *[Most recently / Until last week]*, *[he/she]* worked as a hotel clerk. *[He/she]* has a BA and 5 years of relevant work experience. *[His/her]* most recent wage was $20/hour. *[ [He/she] was laid off in April 2020, and [he/she] has not worked since.* ***/*** *[He/she] was laid off in April 2020, rehired in July 2020, and laid off again in November 2020. [He/she] has not worked since.* ***/*** *[He/she] was employed continuously throughout the pandemic, until the hotel where [he/she] worked went out of business a few days ago.* ***/*** *[He/she] stopped working in April 2020 to supervise online school for [his/her] children, and [he/she] has not worked since. ]* |
| --- | --- | --- |

**SI Table 4.** Distribution of randomly assigned employment histories

|  | **Count** | **Percent** |
| --- | --- | --- |
| **Applicant A** |  |  |
| Continuously unemployed | 231 | 23.7 |
| Yo-yo unemployment | 275 | 28.2 |
| Supervised virtual school | 233 | 23.9 |
| Continuously employed | 235 | 24.1 |
| Total | 974 | 100* |
|  |  |  |
| **Applicant B** |  |  |
| Continuously unemployed | 231 | 23.7 |
| Yo-yo unemployment | 235 | 24.1 |
| Supervised virtual school | 235 | 24.1 |
| Continuously employed | 273 | 28.0 |
| Total | 974 | 100* |
|  |  |  |
| **Applicant C** |  |  |
| Continuously unemployed | 275 | 28.2 |
| Yo-yo unemployment | 233 | 23.9 |
| Supervised virtual school | 232 | 23.8 |
| Continuously employed | 234 | 24.0 |
| Total | 974 | 100* |
|  |  |  |
| **Applicant D** |  |  |
| Continuously unemployed | 242 | 24.9 |
| Yo-yo unemployment | 244 | 25.1 |
| Supervised virtual school | 244 | 25.1 |
| Continuously employed | 244 | 25.1 |
| Total | 974 | 100* |
|  |  |  |
| **Applicant E** |  |  |
| Continuously unemployed | 246 | 25.3 |
| Yo-yo unemployment | 243 | 25.0 |
| Supervised virtual school | 239 | 24.5 |
| Continuously employed | 246 | 25.3 |
| Total | 974 | 100* |

**SI Table 4,** continued.

| **Applicant F** |  |  |
| --- | --- | --- |
| Continuously unemployed | 244 | 25.1 |
| Yo-yo unemployment | 244 | 25.1 |
| Supervised virtual school | 249 | 25.6 |
| Continuously employed | 237 | 24.3 |
| Total | 974 | 100* |
|  |  |  |
| **Across all applicant profiles** |  |  |
| Continuously unemployed | 1,469 | 25.1 |
| Yo-yo unemployment | 1,474 | 25.2 |
| Supervised virtual school | 1,432 | 24.5 |
| Continuously employed | 1,469 | 25.1 |
| Total | 5,844 | 100* |

*Note: Percentages marked with asterisks totaled 100 before rounding.*

**SI Table 5.** Distribution of randomly assigned gender identities

|  | **Count** | **Percent** |
| --- | --- | --- |
| **Applicant A** |  |  |
| Male | 488 | 49.9 |
| Female | 486 | 50.1 |
| Total | 974 | 100 |
|  |  |  |
| **Applicant B** |  |  |
| Male | 483 | 49.6 |
| Female | 491 | 50.4 |
| Total | 974 | 100 |
|  |  |  |
| **Applicant C** |  |  |
| Male | 481 | 49.4 |
| Female | 493 | 50.6 |
| Total | 974 | 100 |
|  |  |  |
| **Applicant D** |  |  |
| Male | 487 | 50.0 |
| Female | 487 | 50.0 |
| Total | 974 | 100 |
|  |  |  |
| **Applicant E** |  |  |
| Male | 483 | 49.6 |
| Female | 491 | 50.4 |
| Total | 974 | 100 |
|  |  |  |
| **Applicant F** |  |  |
| Male | 491 | 49.6 |
| Female | 483 | 50.4 |
| Total | 974 | 100 |
|  |  |  |
| **Across all applicant profiles** |  |  |
| Male | 2,931 | 50.1 |
| Female | 2,913 | 49.9 |
| Total | 5,844 | 100 |

**SI Table 6.** Distribution of randomly assigned racial identities

|  | **Count** | **Percent** |
| --- | --- | --- |
| **Applicant A** |  |  |
| Black | 491 | 50.4 |
| White | 483 | 49.6 |
| Total | 974 | 100 |
|  |  |  |
| **Applicant B** |  |  |
| Black | 483 | 49.6 |
| White | 491 | 50.4 |
| Total | 974 | 100 |
|  |  |  |
| **Applicant C** |  |  |
| Black | 489 | 50.2 |
| White | 485 | 49.8 |
| Total | 974 | 100 |
|  |  |  |
| **Applicant D** |  |  |
| Black | 485 | 49.8 |
| White | 489 | 50.2 |
| Total | 974 | 100 |
|  |  |  |
| **Applicant E** |  |  |
| Black | 492 | 50.5 |
| White | 482 | 49.5 |
| Total | 974 | 100 |
|  |  |  |
| **Applicant F** |  |  |
| Black | 483 | 49.6 |
| White | 491 | 50.4 |
| Total | 974 | 100 |
|  |  |  |
| **Across all applicant profiles** |  |  |
| Black | 2,923 | 50.0 |
| White | 2,921 | 50.0 |
| Total | 5,844 | 100 |

**SI Table 7.** Adjectives provided to survey respondents.

| Lazy |
| --- |
| Family-oriented |
| Hardworking |
| Dedicated |
| Erratic |
| Professional |
| Unmotivated |
| Qualified |
| Unreliable |
| Caring |
| Emotional |

**SI Table 8.** Fig 1 results (Model 1), with robustness checks (Models 2 and 3)

|  | (1) | (2) | (3) |
| --- | --- | --- | --- |
|  | Change in Probability of Hiring | Change in Probability of Hiring | Change in Probability of Hiring |
|  |  |  |  |
| Continuous Unemployment | -0.074*** | -0.073*** | -0.074** |
|  | (0.020) | (0.020) | (0.021) |
| Yoyo Unemployment | -0.076*** | -0.078*** | -0.065** |
|  | (0.020) | (0.020) | (0.021) |
| Supervised Virtual School | -0.073*** | -0.075*** | -0.070** |
|  | (0.020) | (0.020) | (0.021) |
| Applicant B | -0.404*** | -0.404*** |  |
|  | (0.025) | (0.025) |  |
| Applicant C | -0.367*** | -0.367*** |  |
|  | (0.026) | (0.026) |  |
| Applicant D | -0.292*** | -0.292*** |  |
|  | (0.021) | (0.021) |  |
| Applicant E | -0.251*** | -0.252*** |  |
|  | (0.021) | (0.021) |  |
| Applicant F | -0.226*** | -0.225*** |  |
|  | (0.023) | (0.023) |  |
| Black |  | 0.079*** |  |
|  |  | (0.011) |  |
| Female |  | 0.035** |  |
|  |  | (0.007) |  |
| Constant | 0.646*** | 0.589*** | 0.385*** |
|  | (0.020) | (0.022) | (0.013) |
| Observations | 5844 | 5844 | 5844 |

Standard errors in parentheses. OLS regressions with robust standard errors clustered by respondent. The unit of analysis is the applicant profile; the reference category is a continuously employed applicant. The dependent variable is a binary variable indicating whether each applicant profile was selected for hiring. Models 1 and 2 include fixed effects by applicant profile, with "Applicant A" as the reference category. Model 1 is the main results reported in Figure 1. Model 2 adds controls for each applicant's randomly assigned race and gender. Model 3 drops all controls and fixed effects.

* = p<0.05, ** = p<0.01, *** = p<0.001.

**SI Table 9**. Fig 2 results in table form, attributes 1-6

|  | (1) | (2) | (3) | (4) | (5) | (6) |
| --- | --- | --- | --- | --- | --- | --- |
|  | Dedicated | Hardworking | Professional | Qualified | Caring | Family-oriented |
| Continuous Unemployment | -0.261*** | -0.259*** | -0.127*** | -0.067** | -0.037* | 0.048* |
|  | (0.023) | (0.022) | (0.022) | (0.021) | (0.016) | (0.021) |
|  |  |  |  |  |  |  |
| Yoyo Unemployment | -0.209*** | -0.186*** | -0.115*** | -0.071*** | -0.037* | -0.014 |
|  | (0.023) | (0.022) | (0.022) | (0.021) | (0.016) | (0.021) |
|  |  |  |  |  |  |  |
| Supervised Virtual School | -0.129*** | -0.258*** | -0.166*** | -0.100*** | 0.204*** | 0.350*** |
|  | (0.023) | (0.021) | (0.023) | (0.021) | (0.020) | (0.022) |
|  |  |  |  |  |  |  |
| Constant | 0.645*** | 0.739*** | 0.422*** | 0.719*** | 0.222*** | 0.426*** |
|  | (0.021) | (0.019) | (0.021) | (0.019) | (0.017) | (0.021) |
|  |  |  |  |  |  |  |
| Observations | 2922 | 2922 | 2922 | 2922 | 2922 | 2922 |

Standard errors in parentheses. All models are OLS with fixed effects by applicant profile (coefficients not reported) and robust standard errors clustered by respondent. The unit of analysis is the applicant profile; the reference category is a continuously employed applicant. The dependent variables are binary variables indicating whether each profile was described with a given adjective.

* = p<0.05, ** = p<0.01, *** = p<0.001.

**SI Table 10**. Fig 2 results in table form, attributes 7-11

|  | (1) | (2) | (3) | (4) | (5) |
| --- | --- | --- | --- | --- | --- |
|  | Emotional | Unreliable | Erratic | Lazy | Unmotivated |
| Continuous Unemployment | 0.011 | 0.013 | 0.013 | 0.042*** | 0.106*** |
|  | (0.008) | (0.008) | (0.009) | (0.009) | (0.013) |
|  |  |  |  |  |  |
| Yoyo Unemployment | 0.014 | 0.033*** | 0.045*** | 0.013 | 0.052*** |
|  | (0.008) | (0.010) | (0.010) | (0.008) | (0.011) |
|  |  |  |  |  |  |
| Supervised Virtual School | 0.027** | 0.013 | 0.008 | 0.011 | 0.012 |
|  | (0.009) | (0.008) | (0.008) | (0.007) | (0.008) |
|  |  |  |  |  |  |
| Constant | 0.021*** | 0.020** | 0.017* | 0.012 | 0.010 |
|  | (0.006) | (0.007) | (0.007) | (0.006) | (0.007) |
|  |  |  |  |  |  |
| Observations | 2922 | 2922 | 2922 | 2922 | 2922 |

Standard errors in parentheses. All models are OLS with fixed effects by applicant profile (coefficients not reported) and robust standard errors clustered by respondent. The unit of analysis is the applicant profile; the reference category is a continuously employed applicant. The dependent variables are binary variables indicating whether each profile was described with a given adjective.

* = p<0.05, ** = p<0.01, *** = p<0.001.

**SI Table 11.** Figure 3 results in table form

|  | (1) | (2) |
| --- | --- | --- |
|  | Respondents with hiring experience | Respondents with supervisory experience |
| Continuous Unemployment | -0.053+ | -0.070** |
|  | (0.030) | (0.026) |
|  |  |  |
| Yoyo Unemployment | -0.085** | -0.089*** |
|  | (0.030) | (0.026) |
|  |  |  |
| Supervised Virtual School | -0.070* | -0.068** |
|  | (0.031) | (0.026) |
|  |  |  |
| Constant | 0.654*** | 0.653*** |
|  | (0.029) | (0.025) |
|  |  |  |
| Observations | 2676 | 3600 |

Standard errors in parentheses. OLS regressions with fixed effects by applicant (coefficients not reported) and robust standard errors clustered by respondent. The unit of analysis is the applicant profile; the reference category is a continuously employed applicant. The dependent variable is a binary variable indicating whether each applicant profile was selected for hiring.

+ = p<0.1, * = p<0.05, ** = p<0.01, *** = p<0.001.

**SI Table 12**. Fig 4 results in table form, attributes 1-6

|  | (1) | (2) | (3) | (4) | (5) | (6) |
| --- | --- | --- | --- | --- | --- | --- |
|  | Dedicated | Hardworking | Professional | Qualified | Caring | Family-oriented |
| Continuous Unemployment | -0.234*** | -0.239*** | -0.112*** | -0.052* | -0.034 | 0.022 |
|  | (0.029) | (0.028) | (0.027) | (0.025) | (0.020) | (0.026) |
|  |  |  |  |  |  |  |
| Yoyo Unemployment | -0.184*** | -0.160*** | -0.110*** | -0.065** | -0.022 | -0.008 |
|  | (0.028) | (0.027) | (0.027) | (0.025) | (0.020) | (0.025) |
|  |  |  |  |  |  |  |
| Supervised Virtual School | -0.104*** | -0.247*** | -0.172*** | -0.096*** | 0.196*** | 0.353*** |
|  | (0.027) | (0.026) | (0.028) | (0.025) | (0.025) | (0.028) |
|  |  |  |  |  |  |  |
| Constant | 0.639*** | 0.738*** | 0.459*** | 0.738*** | 0.220*** | 0.422*** |
|  | (0.026) | (0.025) | (0.026) | (0.023) | (0.021) | (0.026) |
|  |  |  |  |  |  |  |
| Observations | 1932 | 1932 | 1932 | 1932 | 1932 | 1932 |

Standard errors in parentheses. All models are OLS with fixed effects by applicant profile (coefficients not reported) and robust standard errors clustered by respondent. The unit of analysis is the applicant profile; the reference category is a continuously employed applicant. The dependent variables are binary variables indicating whether each profile was described with a given adjective.

* = p<0.05, ** = p<0.01, *** = p<0.001.

**SI Table 13**. Fig 4 results in table form, attributes 7-11

|  | (1) | (2) | (3) | (4) | (5) |
| --- | --- | --- | --- | --- | --- |
|  | Emotional | Unreliable | Erratic | Lazy | Unmotivated |
| Continuous Unemployment | 0.006 | 0.018 | 0.018 | 0.056*** | 0.113*** |
|  | (0.010) | (0.011) | (0.012) | (0.012) | (0.016) |
|  |  |  |  |  |  |
| Yoyo Unemployment | 0.012 | 0.028* | 0.047*** | 0.012 | 0.063*** |
|  | (0.010) | (0.012) | (0.013) | (0.011) | (0.014) |
|  |  |  |  |  |  |
| Supervised Virtual School | 0.017 | 0.012 | 0.014 | 0.016 | 0.018 |
|  | (0.011) | (0.011) | (0.011) | (0.011) | (0.010) |
|  |  |  |  |  |  |
| Constant | 0.025** | 0.015 | 0.020* | 0.016 | 0.012 |
|  | (0.008) | (0.008) | (0.010) | (0.009) | (0.009) |
|  |  |  |  |  |  |
| Observations | 1932 | 1932 | 1932 | 1932 | 1932 |

Standard errors in parentheses. All models are OLS with fixed effects by applicant profile (coefficients not reported) and robust standard errors clustered by respondent. The unit of analysis is the applicant profile; the reference category is a continuously employed applicant. The dependent variables are binary variables indicating whether each profile was described with a given adjective.

* = p<0.05, ** = p<0.01, *** = p<0.001.

**SI Table 14.** Figure 5 in table form

|  | (1) | (2) |
| --- | --- | --- |
|  | Respondents who lost job  or income due to COVID | Respondents who did not lose job  or income due to COVID |
| Continuous Unemployment | -0.095** | -0.064** |
|  | (0.036) | (0.025) |
|  |  |  |
| Yoyo Unemployment | -0.109** | -0.058* |
|  | (0.035) | (0.025) |
|  |  |  |
| Supervised Virtual School | -0.095** | -0.061* |
|  | (0.034) | (0.025) |
|  |  |  |
| Constant | 0.656*** | 0.641*** |
|  | (0.032) | (0.025) |
|  |  |  |
| Observations | 2088 | 3756 |

Standard errors in parentheses. OLS regressions with fixed effects by applicant (coefficients not reported) and robust standard errors clustered by respondent. The unit of analysis is the applicant profile; the reference category is a continuously employed applicant. The dependent variable is a binary variable indicating whether each applicant profile was selected for hiring.

* = p<0.05, ** = p<0.01, *** = p<0.001.

**SI Table 15.** Figure 6 results in table form

|  | (1) | (2) |
| --- | --- | --- |
|  | Respondents who say COVID  is a serious threat | Respondents who say COVID  is not a serious threat |
| Continuous Unemployment | -0.053* | -0.124*** |
|  | (0.025) | (0.036) |
|  |  |  |
| Yoyo Unemployment | -0.071** | -0.084* |
|  | (0.024) | (0.039) |
|  |  |  |
| Supervised Virtual School | -0.086*** | -0.040 |
|  | (0.024) | (0.037) |
|  |  |  |
| Constant | 0.632*** | 0.675*** |
|  | (0.023) | (0.036) |
|  |  |  |
| Observations | 4062 | 1782 |

Standard errors in parentheses. OLS regressions with fixed effects by applicant (coefficients not reported) and robust standard errors clustered by respondent. The unit of analysis is the applicant profile; the reference category is a continuously employed applicant. The dependent variable is a binary variable indicating whether each applicant profile was selected for hiring.

* = p<0.05, ** = p<0.01, *** = p<0.001.

**SI Table 16.** Figure 7 results in table form

|  | (1) | (2) |
| --- | --- | --- |
|  | Democratic  respondents | Republican  respondents |
| Continuous Unemployment | -0.040 | -0.096** |
|  | (0.031) | (0.035) |
|  |  |  |
| Yoyo Unemployment | -0.061* | -0.104** |
|  | (0.030) | (0.035) |
|  |  |  |
| Supervised Virtual School | -0.044 | -0.085* |
|  | (0.030) | (0.035) |
|  |  |  |
| Constant | 0.643*** | 0.639*** |
|  | (0.029) | (0.034) |
|  |  |  |
| Observations | 2556 | 2010 |

Standard errors in parentheses. OLS regressions with fixed effects by applicant (coefficients not reported) and robust standard errors clustered by respondent. The unit of analysis is the applicant profile; the reference category is a continuously employed applicant. The dependent variable is a binary variable indicating whether each applicant profile was selected for hiring.

* = p<0.05, ** = p<0.01, *** = p<0.001.

**SI Table 17.** Figure 8 results in table form

|  | (1) | (2) | (3) |
| --- | --- | --- | --- |
|  | Liberal respondents | Moderate  respondents | Conservative respondents |
| Continuous Unemployment | -0.050 | -0.085* | -0.085** |
|  | (0.036) | (0.039) | (0.031) |
|  |  |  |  |
| Yoyo Unemployment | -0.059 | -0.102** | -0.073* |
|  | (0.036) | (0.037) | (0.033) |
|  |  |  |  |
| Supervised Virtual School | -0.048 | -0.100** | -0.076* |
|  | (0.035) | (0.038) | (0.032) |
|  |  |  |  |
| Constant | 0.643*** | 0.686*** | 0.621*** |
|  | (0.034) | (0.037) | (0.031) |
|  |  |  |  |
| Observations | 1782 | 1656 | 2406 |

Standard errors in parentheses. OLS regressions with fixed effects by applicant (coefficients not reported) and robust standard errors clustered by respondent. The unit of analysis is the applicant profile; the reference category is a continuously employed applicant. The dependent variable is a binary variable indicating whether each applicant profile was selected for hiring.

* = p<0.05, ** = p<0.01, *** = p<0.001.
