# Supplementary material for: Perceptions of pandemic resume gaps: Survey experimental evidence from the United States
Source: PLoS One. 2023 Mar 16;18(3):e0281449. doi: 10.1371/journal.pone.0281449 (PMC10019729; doi:10.1371/journal.pone.0281449)
Supplement: Attachment — Submitted filename: Response Memo.docx [file pone.0281449.s003.docx]

Thank you for this opportunity to revise my manuscript, and thank you for the valuable feedback. I have made multiple significant changes to the manuscript, and I hope the new draft meets with your approval.

While I appreciate the advice of both reviewers and the editor, I would particularly like to thank R2 for their exceptionally thorough and helpful review. R2's analysis and advice are spot-on, and I have implemented all the changes they recommended. I believe these efforts have significantly improved the manuscript, resulting in a more appropriate framing, better engagement with the literature, a stronger theoretical foundation, and more attention to studies from other countries.

In line with the recommendations of the reviewers and the editor, I have made multiple major changes to the manuscript, including:

-reframing and rewriting the paper (including a new title!)

-incorporating new literature, doubling the number of references in the manuscript, and adding studies and data from multiple other countries

-expanding, clarifying, and placing greater emphasis on the discussion of external validity

Below, I describe these major changes, then I respond to additional points raised by each reviewer.

**Major Changes**

1. Reframing and reorienting the manuscript

I agree with R2's observation that because the survey experiment was conducted with a nationally representative sample of US adults, it is primarily a study of public opinion toward those who were out of work during the COVID-19 pandemic. As R2 correctly points out, "the paper provides mainly evidence for perceptions of job-seekers in the general population." R2 therefore suggests that I either reframe the paper to focus exclusively on respondents with hiring experience, or to reframe the paper to be about "perceptions of the general population."

In this revision, I have made major changes to emphasize that this is primarily a study of public attitudes toward the unemployed. I changed the title, rewrote the abstract, and edited the language throughout the paper to place greater emphasis on perceptions, rather than actual hiring of job applicants. For example, I have greatly reduced the use of the term "job-seeker." I also reduced the use of the terms "hiring" and "hiring decision" when discussing the survey experiment. Instead, in this draft, I describe how respondents selected, chose, or preferred fictional applicants with different employment histories. I believe this is more accurate and transparent, and this language is more consistent with what is actually happening in the survey experiment.

In the new introduction, I situate the paper in relation to other studies of perceptions of the unemployed, and I articulate why public opinion toward the unemployed matters (see lines 93-105). I explain that public opinion shapes public policy, and stigma has multiple negative impacts on unemployed individuals. For instance, through "stigma consciousness" (Gurr and Jungbauer-Gans 2013), merely being aware of others' judgments can affect job search behavior and outcomes (Krug, Drasch, and Jungbauer-Gans 2019). While multiple prior studies have examined public perceptions of the unemployed in other contexts (Maassen and de Goede 1991, Eardley and Matheson 2000, Furåker and Blomsterberg 2003, Buffel and Van de Velde 2019), this is (to the best of my knowledge), to the best of my knowledge this manuscript is the first research to offer insights into perceptions of individuals who were out of work at the height of the COVID-19 pandemic.

2. Expanded engagement with the literature & more theory

I would like to thank R2 for the extensive list of recommended references. I have cited all of R2's recommended references into this draft of the manuscript. In addition, I have added more than 20 other references to give the paper a better theoretical grounding, to make its contributions and limitations clearer, and to better situate it in an international context.

Equipped with this additional research and reading, I have also revised the manuscript to clarify the theoretical interpretation of my results. I agree with R2 that this is really a study about unemployment stigma, not unemployment scarring (thank you, R2, for pointing that out!). I now discuss stigma at multiple points in the paper. I also argue that signaling is the most likely mechanism for the results of the experiment. The paper now includes citations to the literature on the negative signaling effects of periods of unemployment (Spence 1973, Vishwanath 1989) and frequent job changes (Cohn et al 2021).

In addition, as recommended by R2, the paper now includes a substantial discussion of differences with prior audit studies on unemployment duration and callback rates for job interviews. This is primarily found on lines 291-308, though I also edited the introduction to clarify that the results in this literature mixed (see lines 70 and 77-78).

Finally, addressing the editor's request for more discussion of studies from other countries, the revised manuscript includes new references to data and research from Japan, Britain, Germany, Switzerland, Sweden, Australia, the Netherlands, Mexico, and a multi-country study conducted across Europe.

3. More emphasis on external validity

R1, R2, and the editor all raised concerns about external validity. I take that feedback seriously, and I have tried to reframe the manuscript to present the results more appropriately.

In this revision, I have not used the results from respondents with hiring and managerial experience as the baseline models (although the editor recommended that I do so). I did not do this because doing so would contravene my pre-registered pre-analysis plan, and because it would be very unusual to present the results for a sub-group of respondents without first presenting the results for the full sample of respondents.

However, I have taken taken multiple steps to expand and place greater emphasis on the results for the respondents with hiring and managerial experience. First, I moved this section up in the manuscript, so it appears more prominently at an earlier point in the manuscript. Second, I added a new figure (Figure 4) that shows how respondents with hiring and managerial experience describe the fictional job applicants. Third, the revised manuscript includes additional discussion of external validity on lines 280-290 and 305-308.

I have made best efforts to address R1's concerns about external validity, and I agree that we should always be careful about generalizing from survey experiments—as I note in the abstract (line 48) and multiple times in manuscript on (lines 284 and 372-74). However, despite their limitations, PLOS One regularly publishes survey experiments on a wide range of topics, including perceptions of COVID-19 policies (Zhang et al 2020), trust in government (Martin et al 2020), discrimination in the housing market (Ghekiere et al. 2022), choices among different COVID-19 vaccines (Kreps and Kriner 2022), pandemic-induced racial and ethnic prejudice (Kaushal, Lu, and Huang 2022), discrimination toward literary authors (Weinberg and Kapelner 2022), and evaluations of restaurants (Maezawa and Kawahara 2021). With the improvements in this draft, I hope this manuscript can eventually be published as well.

**Additional Responses to Reviewers**

R1

I would like to thank R1 for their attention to the manuscript.

In this revision, I have adopted several of the changes recommended by R1. First, I have improved the theoretical background for the paper. Second, I have described the results as positive discrimination at several points (including lines 38-39 in the abstract and lines 86-87 in the body of the manuscript). Thank you for this helpful suggestion.

I also appreciate R1's interest in analyzing the age and health status of the fictional job applicants, but I was not able to act on this request because age was held constant in the experimental vignettes and the profiles did not include any information about the fictional applicants' health status (as clarified in SI Tables 12-13).

R2

I am deeply grateful for R2's comprehensive, constructive review, which was instrumental in informing my revisions. I also appreciate R2's praise for the manuscript's "great design and analysis."

In response to R2's first main concern, I re-oriented most of the paper to focus more on public opinion toward individuals who were unemployed during the pandemic. However, I also partially adopted R2's second recommended strategy, which was to focus more on the results from respondents with hiring experience. That expanded discussion now appears more prominently in the manuscript, starting on line 233.

In the expanded section on the results for respondents with hiring and managerial experience, I also address R2's concern about statistical significance of the coefficient on continuous unemployment. As noted in the manuscript, the standard errors are larger for that analysis, because the N is smaller (Lines 240-242). The p-value for that coefficient is 0.08, which is above the conventional 0.05 threshold but under 0.1. I acknowledge this in the manuscript and describe that result as only marginally statistically significant (Lines 257-258). If I were to combine the respondents with hiring experience together with the respondents with managerial experience (to create a larger N), this result would be statistically significant with p<0.05. However, I did not make this change in the manuscript because it would amount to p-hacking, which is not a good research practice. I hope R2 understands this rationale.

In addition, I revised the title to remove the term "unemployment scarring," and I removed most discussions of "scarring" from the paper. I would like to thank R2 for pointing out this problem, and for advising me on the correct term ("unemployment stigma").

R2's literature references, the suggestion to consider signaling theory, and the recommendation to engage more with audit studies were all extremely helpful. Thank you so much!

R2 went above and beyond in writing their review, contributing substantially to my revisions. Thank you again, I hope you enjoy reading this new version of the manuscript!

**WORKS CITED**

Buffel V, Van de Velde S. Comparing negative attitudes toward the unemployed across European countries in 2008 and 2016: The role of the unemployment rate and job insecurity. International Journal of Public Opinion Research. 2019;31(3):419-440.

Cohn A, Maréchal MA, Schneider F, Weber RA. Frequent job changes can signal poor work attitude and reduce employability. Journal of the European Economic Association. 2021;19(1):475-508.

Eardley T, Matheson G. Australian attitudes to unemployment and unemployed people. Australian Journal of Social Issues. 2000;35(3): 181-202.

Furåker B, Blomsterberg M. Attitudes towards the unemployed: An analysis of Swedish survey data. International Journal of Social Welfare. 2003;12:193-203.

Ghekiere A, Verhaeghe P, Baert S, Derous E, Schelfhout S. Introducing a vignette experiment to study mechanisms of ethnic discrimination on the housing market. PLoS One 2022;17(10): e0276698. https://doi.org/10.1371/journal.pone.0276698

Gurr T, Jungbauer-Gans M. Stigma consciousness among the unemployed and prejudices against them: Development of two scales for the 7th wave of the panel study, "Labour Market and Social Security (PASS)." Journal for Labour Market Research. 2013;46:335-351.

Kaushal N, Lu Y, Huang X. Pandemic and prejudice: Results from a national survey experiment." PLOS One. 2022;17(4): e0265437. https://doi.org/10.1371/journal.pone.0265437

Kreps S, Kriner DL. Communication about vaccine efficacy and COVID-19 vaccine choice: Evidence from a survey experiment in the United States. PLoS One. 2022; 17(3): e0265011. https://doi.org/10.1371/journal.pone.0265011

Krug G, Drasch K, Jungbauer-Gans M. The social stigma of unemployment: Consequences of stigma consciousness on job search attitudes, behaviour and success. Journal for Labour Market Research. 2019;53(11):1-27.

Maassen G, de Goede M. Changes in public opinion on the unemployed: The case of the Netherlands. International Journal of Public Opinion Research. 1991;3(2):182-194.

Maezawa T, Kawahara JI. A label indicating an old year of establishment improves evaluations of restaurants and shops serving traditional foods. PLoS One*.* 2021;16(11): e0259063. https://doi.org/10.1371/journal.pone.0259063

Martin A, Orr R, Peyton K, Faulkner N. Political Probity Increases Trust in Government: Evidence from Randomized Survey Experiments. PLoS One. 2020;15(2): e0225818. https://doi.org/10.1371/journal.pone.0225818

Spence M. Job market signaling. The Quarterly Journal of Economics 1973;87(3):355-374.

Vishwanath T. Job search, stigma effect, and escape rate from unemployment. Journal of Labor Economics. 1989;7(4):487-502.

Weinberg, DB, Kapelner A. 2022Do book consumers discriminate against Black, female, or young authors?" PLoS One. 2022;17(6): e0267537. https://doi.org/10.1371/journal.pone.0267537

Zhang B, Kreps S, McMurray N, McCain RM. Americans' perceptions of privacy and surveillance in the COVID-19 pandemic. PLOS One. 2020;15(12): e0242652. https://doi.org/10.1371/journal.pone.0242652
