# Supplementary material for: Perceptions of pandemic resume gaps: Survey experimental evidence from the United States
Source: PLoS One. 2023 Mar 16;18(3):e0281449. doi: 10.1371/journal.pone.0281449 (PMC10019729; doi:10.1371/journal.pone.0281449)
Supplement: Attachment — Submitted filename: Bateson 2nd Revision memo.docx [file pone.0281449.s004.docx]

To the editor and reviewers:

Thank you for the invitation to revise and resubmit my manuscript. Once again, I would like to thank R2 for their detailed comments and constructive advice. Additionally, I appreciate the queries and suggestions from the editor.

In this revision, I have made multiple changes to address the editor's comments and questions.

There are 5 improvements in this revision:

1. Better explanation of the randomization in the survey experiment

The editor raised a number of questions about the randomization in the survey experiment. I agree that the randomization process was not thoroughly explained in the prior drafts; thank you for drawing my attention to this issue.

For this revision, I created two new tables (Table 1 and Table 2 on pp. 6-7 of the manuscript). These tables summarize the characteristics that were held constant and and the characteristics that were randomized within each applicant profile.

I hope Tables 1 and 2 clarify that there were only 3 characteristics randomized within the applicant profiles: pandemic employment history, race, and gender. I also added new text describing the randomization process (see lines 139-143 on p. 5).

Additionally, in the captions for Tables 2 and 3, I explain that this is a 2 x 2 x 4 randomization, resulting in 16 different versions of each applicant profile.

I hope this additional information better explains what was held constant, and what was randomized.

2. More information about the distribution of randomized characteristics

The editor also requested additional information about the distribution of the randomized characteristics across the applicant profiles.

In the SI, I have added 3 new tables summarizing the counts and percentages of the employment histories and racial and gender identities assigned to each fictional applicant profile. These are SI Tables 4-6, which are mentioned in lines 142-143 on p. 5 of the manuscript.

The randomization was programmed using the "evenly present elements" function in Qualtrics, and overall it was quite well-balanced. However, as noted in SI Tables 4-6, within some applicant profiles, there are a few slight imbalances. I believe this may have resulted from the attrition of a small number of survey respondents, or from the variation inherent in the randomization process.

However, even if we control for the race and gender assigned to each applicant profile, the impact of a pandemic resume gap remains negative and statistically significant (see SI Table 3, model 2).

3. Additional robustness checks

As noted above, I have revised SI Table 8, which reports the main results from Fig 1 in the manuscript.

Now SI Table 8 includes two new robustness checks (see models 2 and 3). Model 2 adds controls for the race and gender randomly assigned to each applicant profile, and Model 3 drops the fixed effects by applicant profile and the controls for race and gender. Across all these models, the coefficients on the pandemic employment histories are negative, statistically significant, and similar in magnitude.

4. Better explanation of the fixed effects by applicant profile

To better explain the fixed effects by applicant profile, I have added new text on lines 188-191 of the manuscript (pp. 8-9).

In short, Applicants A, B, C, D, E, and F each have slightly different biographies, with multiple characteristics that are fixed (not randomized) throughout the experiment, such as their age, number of children, level of education, years of work experience, most recent job title, and most recent wage. In addition, the applicants are labelled A, B, C, D, E, and F. These differences could make certain applicants seem more appealing, regardless of their employment histories. That is why I use fixed effects by applicant profile in my main models. The fixed effects by applicant profile control for the differences across the applicant profiles, allowing us to isolate the effect of the randomly assigned employment histories.

As recommended by the editor, I am now reporting the coefficients for the fixed effects by applicant in SI Table 8. I did not add the coefficients for the fixed effects to all the subsequent tables, because it would make all the tables much longer, and the coefficients for each applicant profile don't really convey any substantively interesting information. However, I am open to reporting the coefficients for the fixed effects in all tables if the editor thinks it is essential.

5. Re-organizing and re-numbering the SI tables

Finally, I have acted on the editor's request to re-number and re-organize the SI tables. There are now 17 tables in the SI, and they appear in the order that they are mentioned in the manuscript.

I hope these changes meet with your satisfaction, and I look forward to hearing back from you soon. Thank you again for considering my work, and thank you for your detailed feedback and guidance.
