# Supplementary material for: Perceptions of pandemic resume gaps: Survey experimental evidence from the United States
Source: PLoS One. 2023 Mar 16;18(3):e0281449. doi: 10.1371/journal.pone.0281449 (PMC10019729; doi:10.1371/journal.pone.0281449)
Supplement: Attachment — Submitted filename: Referee Report Corona Pandemic Effects on Unemployment.pdf [file pone.0281449.s002.pdf]

Referee Report „Unemployment scarring and the COVID-19 pandemic: How pandemic resume gaps affect perceptions of job-seekers"

The paper provides a survey experiment on the signalling effect of resume gaps during the COVID-19 pandemic. The paper provides evidence for the negative effect of different resume gaps, indicating the potential negative consequences of the pandemic for future labor market outcomes. This original research tries to fill an important current gap in the literature on the negative consequences of unemployment during the pandemic.

I have two main concerns and one minor point for the current version of the manuscript. While they imply a major revision of the manuscript, given the great design and analysis, I have no doubt that the revision is feasible and would make a great future publication in PLOS ONE. This is why I recommend a revise and resubmit.

Main Concern 1:

The paper conducts a survey experiment on a national representative sample of the United States. However, the general population is not suitable to make implications about negative consequences during the hiring process. The paper provides mainly evidence for perceptions of job-seekers in the general population, not what is observable during the hiring process. While the perceptions of the general population are of importance for political interventions, a survey experiment on negative consequences in the labor market should focus on decision makers in the hiring process. I therefore welcome the robustness check which focuses on individuals with experience in the hiring process. Yet, the results focusing on this subgroup (Figure 7 Panel a)) provides no statistical significant evidence for negative consequences for continuous unemployment.

Suggestion:

I therefore recommend deciding to focus on individuals with hiring experience (which is common practice in such survey experiments) or to reframe the paper in perceptions of the general population. This is of importance, given the fact that the results are not in line with previous audit studies on the impact of the unemployment duration during the hiring process for the United States.

Main Concern 2:

My second main concern is related to the literature work of the manuscript, which does not cover a substantial literature relevant for the research question. In the following I give an overview of this literature and show why which papers are of relevance.

Motivated by the Great Recession, there is a growing literature of audit studies that look at the effect of the unemployment duration for the job finding rates. The advantage of these papers compared to survey experiments is that they allow to avoid demand effects. 3 out of 5 such audit studies do not find discrimination against long term unemployed (Farber et al., 2016; Farber et al., 2017; Nunley et al., 2017). The remaining two papers find evidence for negative effects during the hiring process, under specific circumstances (Ghayad, 2013; Kroft et al.,

2013). Given that the findings stand in contrast with most of this literature, I would at least want to see a discussion that tries to explain these differences.

There are further paper for the European context, which might be of relevance for different reasons (Cohn et al., 2021; Eriksson and Rooth, 2014; Nüß, 2018; Oberholzer-Gee, 2018).

The paper needs a theoretical foundation why unemployment during the pandemic should effect labor market outcomes at al. Given the findings, the perceived lack of motivation indicates that the signalling theory is a good starting point, also supported by previous experiments (Oberholzer-Gee, 2008; Kroft et al., 2013).

The paper by Cohn et al. (2021) provides a laboratory experiment, a survey experiment as well as audit study and shows that yo-yo unemployment is interpreted as a signal for low reliability which leads to worsen labor market outcomes. This paper is closest to the manuscript and should be therefore considered. This might also help to bring the manuscript more in context of the literature.

Lastly, two papers consider business cycle effects of unemployment spells (Korft et al., 2013; Nüß, 2018). These experiments show that unemployment hast only negative effects on job findings in strong labor markets (i.e. low unemployment). In line with the signalling theory, unemployment spells are interpreted as a negative signall when unemployment is low and individuals should easily find a job. If people still do not find a job, employers get sceptical about the productivity and motivation of the applicants. In contrast, when unemployment is high and it is more difficult to find a job, employers interpret less into the unemployment. Given the fact that the results of the survey experiment find negative effects during the pandemic, independent of the reason for the unemployment spell, the manuscript should at least discuss its findings in context of this literature.

Minor Concern:

A last minor point is related to the title, which covers the term “Unemployment scar”. An unemployment scar, is the long run effect of long time past unemployment on current labor market outcomes. In contrast, negative consequences of recent/current unemployment is named an unemployment stigma (Vishwanath, 1989).

Suggestion:

So to analyse an unemployment scar in a survey experiment would mean to provide participants information about past unemployment. It can be ruled out that findings of survey experiments and audit studies are due to an unemployment scar because Eriksson and Rooth (2014) tested current and past unemployment experience in an audit study, providing evidence for discrimination based on current unemployment spells, while past unemployment spells had no effect. So technically calling it a scar is the wrong term and it would be great to be more precise with it.

References:

Cohn, A., Maréchal, M. A., Schneider, F., & Weber, R. A. (2021). Frequent job changes can signal poor work attitude and reduce employability. Journal of the European Economic Association, 19(1), 475-508.

Eriksson, S., & Rooth, D. O. (2014). Do employers use unemployment as a sorting criterion when hiring? Evidence from a field experiment. American economic review, 104(3), 1014-39.

Farber, H. S., Silverman, D., & Von Wachter, T. (2016). Determinants of callbacks to job applications: An audit study. American Economic Review, 106(5), 314-18.

Farber, H. S., Silverman, D., & Von Wachter, T. M. (2017). Factors determining callbacks to job applications by the unemployed: An audit study. RSF: The Russell Sage Foundation Journal of the Social Sciences, 3(3), 168-201.

Ghayad, R. (2013). The jobless trap. Northeastern University.

Kroft, K., Lange, F., & Notowidigdo, M. J. (2013). Duration dependence and labor market conditions: Evidence from a field experiment. The Quarterly Journal of Economics, 128(3), 1123-1167.

Nunley, J. M., Pugh, A., Romero, N., & Seals, R. A. (2017). The effects of unemployment and underemployment on employment opportunities: Results from a correspondence audit of the labor market for college graduates. Ilr review, 70(3), 642-669.

Nüß, P. (2018). Duration dependence as an unemployment stigma: Evidence from a field experiment in Germany (No. 2018-06). Economics Working Paper.

Oberholzer-Gee, F. (2008). Nonemployment stigma as rational herding: A field experiment. Journal of Economic Behavior & Organization, 65(1), 30-40.

Vishwanath, T. (1989). Job search, stigma effect, and escape rate from unemployment. Journal of Labor Economics, 7(4), 487-502.
